# COVID-19 and Cardiovascular Diseases: From Cellular Mechanisms to Clinical Manifestations

**DOI:** 10.14336/AD.2023.0314

**Published:** 2023-12-01

**Authors:** Hongyang Shu, Zheng Wen, Na Li, Zixuan Zhang, Bala Musa Ceesay, Yizhong Peng, Ning Zhou, Dao Wen Wang

**Affiliations:** ^1^Division of Cardiology, Department of Internal Medicine, Tongji Hospital, Tongji Medical College, Huazhong University of Science and Technology, Wuhan 430000, China.; ^2^Hubei Key Laboratory of Genetics and Molecular Mechanism of Cardiologic Disorders, Huazhong University of Science and Technology, Wuhan 430000, China.; ^3^Department of Orthopedics, Union Hospital, Tongji Medical College, Huazhong University of Science and Technology, Wuhan 430000, China

**Keywords:** COVID-19, cardiovascular diseases, Long-COVID, vaccines

## Abstract

Coronavirus disease 2019 (COVID-19) caused by the severe acute respiratory syndrome coronavirus 2 (SARS-CoV-2), quickly spread worldwide and led to over 581 million confirmed cases and over 6 million deaths as 1 August 2022. The binding of the viral surface spike protein to the human angiotensin-converting enzyme 2 (ACE2) receptor is the primary mechanism of SARS-CoV-2 infection. Not only highly expressed in the lung, ACE2 is also widely distributed in the heart, mainly in cardiomyocytes and pericytes. The strong association between COVID-19 and cardiovascular disease (CVD) has been demonstrated by increased clinical evidence. Preexisting CVD risk factors, including obesity, hypertension, and diabetes etc., increase susceptibility to COVID-19. In turn, COVID-19 exacerbates the progression of CVD, including myocardial damage, arrhythmia, acute myocarditis, heart failure, and thromboembolism. Moreover, cardiovascular risks post recovery and the vaccination-associated cardiovascular problems have become increasingly evident. To demonstrate the association between COVID-19 and CVD, this review detailly illustrated the impact of COVID-19 on different cells (cardiomyocytes, pericytes, endothelial cells, and fibroblasts) in myocardial tissue and provides an overview of the clinical manifestations of cardiovascular involvements in the pandemic. Finally, the issues related to myocardial injury post recovery, as well as vaccination-induced CVD, has also been emphasized.

The emergence of coronavirus disease 2019 (COVID-19) has overwhelmed the medical systems of several countries and posed unprecedented challenges to the mankind [1]. The high infectivity and stealth of SARS-CoV-2 allows it to spread rapidly [2-4]. As of August 1, 2022, SARS-CoV-2 has spread to more than 200 countries. Although dyspnea is the major symptom of SARS-CoV-2 [5], cardiovascular damages, such as acute myocardial injury [5], heart failure [6], and cardiogenic shock [7, 8], not only occur during the acute phase of infection but also persist in the recovery phase [9]. In addition, patients with cardiovascular diseases (CVDs) and risk factors are highly susceptible to COVID-19 and present severe clinical manifestations and poor prognosis post infection.

Compared to the previous two coronaviruses of the century (SARS-CoV and Middle East respiratory syndrome coronavirus [MERS-CoV]), SARS-CoV-2 displays considerably higher transmission rate and death toll and exerts pronounced effects on the cardiovascular system, in spite of the relatively lower overall mortality rate [10]. The first known case of severe acute respiratory syndrome (SARS) occurred in Foshan, China, in November 2002. With a mortality rate of approximately 10% [10], 8,096 cases and 774 deaths were reported in 27 countries by July 2003. In June 2012, 10 years after the first appearance of SARS-CoV, MERS-CoV emerged in the Arabian Peninsula [11], with a mortality rate of approximately 35%, infecting more than 2,000 people across 27 countries. The cases included occasional reports of cardiovascular complications [12]. SARS-CoV-2 was first reported in Wuhan, China in December 2019 [13] and has since infected more than 500 million people, with an overall mortality rate of approximately 4% [14]. Approximately 62% of the patients hospitalized with COVID-19 have been reported to have acute myocardial injuries, with the severity of the injury being closely related to mortality [15-20].

This review focuses on the effects of SARS-CoV-2 on the cardiovascular system. We reviewed the evidence and mechanisms of different cellular injuries in myocardial tissues and the clinical manifestations of cardiovascular involvement and discussed issues related to myocardial injury in the recovery stage and after vaccination.

## 1. CVDs and COVID-19

**Figure 1.**
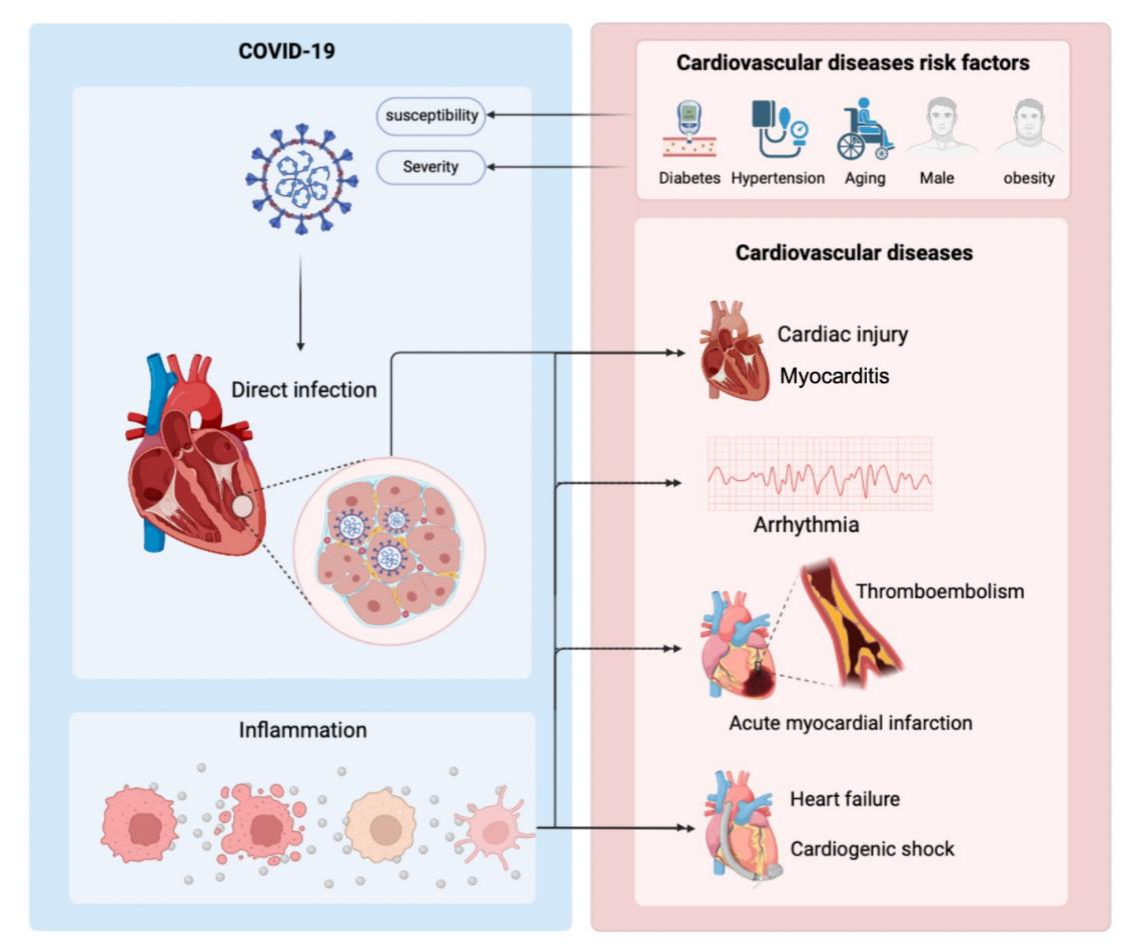
**People with cardiovascular risk factors (such as aging, obesity, hypertension, male, and diabetes) have increased susceptibility to COVID-19 and display severe symptoms**. SARS-CoV-2 has the potential to cause damage to the heart through direct infection and inflammation, manifested as acute myocardial injury, acute myocardial infarction, heart failure, myocarditis, cardiogenic shock, and thromboembolism.

Hypertension and other CVDs are among the most commonly reported complications among patients hospitalized with COVID-19 [21]. Troponin elevation and coronary artery disease history are predictive factors of the severity or mortality of COVID-19 [18]. By August 2020, Australia and New Zealand jointly declared that pre-existing CVDs could increase the incidence rate and mortality of COVID-19. The existence of potential CVD increases the case fatality rate of COVID-19 by 5-10 times [22]. SARS-CoV-2 causes a variety of cardiovascular diseases, including myocardial injury, arrhythmia, myocardial infarction, and heart failure, among others, through direct damage to myocardial tissues and indirect inflammatory storm. This section summarizes the incidence rate and discusses the possible pathophysiological mechanism of several common cardiovascular diseases in patients with COVID-19 (Fig.1).

### 1.1 Acute cardiac injury and COVID-19

Acute cardiac injury is defined as the elevation of cardiac troponin levels above the 99^th^ percentile reference upper limit [23]. The prevalence of acute myocardial injury in patients hospitalized with COVID-19 has been globally reported to be in the ranges 6.9-36%: Turkey, 6.9%; China, 12.4-19.7%; Spain, 26.9%; Italy, 27%; United States, 36% [24-27]. Compared with patients without myocardial injury, patients with myocardial injury have more electrocardiogram abnormalities, higher inflammatory biomarkers, and an increased incidence of major echocardiographic abnormalities, including left ventricular wall motion abnormalities, global left ventricular function disorders, left ventricular diastolic dysfunction grade II or III, right ventricular dysfunction, and pericardial effusion [16]. Patients with cardiac injury show further increased complications than those without; these complications include acute respiratory distress syndrome, acute kidney injury, electrolyte disturbances, hypoalbuminemia, and coagulation disorders [19]. Mortality rates in patients with heart damage are much higher than those in patients without heart damage [19, 28]. Non-survivors were reported to have a significantly higher proportions of elevated troponin than the survivors [29]. Mortality in patients with COVID-19 with troponin levels below the upper limit of normal was 22.7%, while that in patients with troponin levels >010 times the upper limit of normal (P < 0.001) was 61.5% [30]. Troponin acts as an independent predictor of mortality in patients with COVID-19; thus, greater the increase in troponin, higher the mortality rate [27].

Despite the severity of effects of acute myocardial injury during the recovery period of severe COVID-19 infection, the cardiac functions of most patients returned to normal; 89% of the patients had a left ventricular ejection fraction of approximately 67%, while 26% of the patients developed myocarditis-like scars. Moreover, myocarditis-like damages were limited to three or fewer myocardial segments [31].

### 1.2 Arrhythmia and COVID-19

Arrhythmia is a COVID-19 associated cardiovascular complication, with approximately 7.3% patients complaining of palpitations as the first symptoms. The overall incidence of arrhythmia in patients with COVID-19 is 16.8%, of which approximately 8.2% constitute atrial arrhythmias (atrial fibrillation or atrial flutter), 10.8% conduction disorders, 8.6% ventricular tachycardia (ventricular tachycardia, tachycardia/ventricular flutter/ ventricular fibrillation), and 12% unclassified arrhythmias [32]. Patients admitted to the intensive care unit (ICU) reported higher rates of arrhythmias than those who were not admitted. Malignant arrhythmia has been a frequent occurrence in patients with COVID-19 than that in the survivors [33]. Fatal tachyarrhythmia always occurs in the presence of severe metabolic imbalance. The incidence of arrhythmias in patients with COVID-19 has been reported to be higher than that in patients with other community-acquired pneumonia (16.8% vs. 4.7%, 95% CI=2.4-8.9), along with higher mortality rates [34]. Approximately one in five people with COVID-19 died after developing cardiac arrhythmia. The mechanism of arrhythmia may be explained by COVID-19-induced metabolic disturbances, hypoxia, neurohormonal and inflammatory stress, and cardiac damage. In addition, QT prolongation caused by certain anti-COVID-19 drugs. such as hydro-xychloroquine, can lead to polymorphic ventricular tachycardia in the form of *torsades de pointes* and sudden cardiac death [35]. In 2020, the Canadian Heart Rhythm Society issued a statement calling for the discontinuation of unnecessary drugs that may increase the QT interval [36].

Notably, approximately 10% of patients with COVID-19 develop post-acute COVID-19 syndrome after recovery, and approximately 25-50% of them have tachycardia or palpitations, which mainly manifest as orthostatic tachycardia syndrome or sinus tachycardia [37]. Recently, Ståhlberg et al suggested that persistent tachycardia may be a subsyndromic or a specific phenotype of acute post-COVID-19 syndrome and recommended labelling it as “post-COVID-19 tachycardia syndrome” [38]. The pathogenesis of this syndrome may be related to excessive inflammation [39], a hypercoagulable state of thrombosis, dysfunction of the renin-angiotensin-aldosterone system, anxiety, depression, and neuroinflammation [40, 41]. However, given the lack of relevant basic research and clinical data on the disease, the pathogenesis of post-COVID-19 tachycardia syndrome remains unclear.

### 1.3 Acute myocardial infarction and COVID-19

Acute myocardial infarction is myocardial necrosis caused by acute and persistent ischemia of the coronary arteries, which can be diagnosed based on typical clinical symptoms, changes in 12-lead electrocardiograms, and myocardial enzymes. ST-T abnormality is the most common electrocardiogram feature among patients with COVID-19, accounting for 40% of the cases [42]. Self-controlled case series and matched cohort studies conducted in Sweden have suggested that COVID-19 is a risk factor for acute myocardial infarction [43]. In a multicenter retrospective study from four countries, only 78 patients with COVID-19 reportedly developed myocardial infarction [44]. In a comprehensive international survey of 1,216 patients with COVID-19 from 69 countries from six continents, 36 patients (3%) were diagnosed with myocardial infarction using echocardiography [45]. Angina may present with dyspnea in case of acute myocardial infarction; approximately 80% of patients with COVID-19 diagnosed with ST-segment elevation myocardial infarction display typical chest pain with or without dyspnea, whereas more than 20% report dyspnea without chest pain [45]. In the context of the COVID-19 pandemic, respiratory diseases associated with dyspnea symptoms often receive special attention, whereas the associated cardiovascular diseases are often underestimated. Mele et al. recently reported a patient with COVID-19, diagnosed by a chest CT scan, with an acute myocardial infarction, reemphasizing the concern for patients with dyspnea-type acute myocardial infarction. The reason of acute myocardial infarction secondary to COVID-19 infection may be that inflammation related to acute infection leads to the formation of thrombus *in situ* or the displacement of pre-existing atherosclerotic plaque.

Notably, approximately 40% of patients with COVID-19 with typical myocardial infarction manifestations are free of cardiomyopathy, possibly owing to increased myocardial demand under inflammatory conditions that lead to type 2 myocardial infarction.

### 1.4 Acute myocarditis (AM) and COVID-19

AM is a disease mainly manifested by localized or diffuse inflammatory lesions in the myocardium [46]. AM is common in viral infections [47]. For example, virus-associated myocarditis was reported during the 1917 influenza pandemic and in the 2002 SARS outbreak [48]. Autopsy demonstrated the presence of viral RNA in the myocardium of 35% of the virus-infected patients [49]. Acute myocarditis was also reported during the outbreak of MERS-CoV [12]. AM is a rare cardiovascular complication of COVID-19 and is estimated to occur in 2.4-4.1 out of 1,000 patients hospitalized with COVID-19 [50]. Pneumonia can also accelerate disease progression. At 120 days, the overall mortality in patients with pneumonia has been observed to be 6.6-15.1% and that in patients without pneumonia is 0% (P = 0.044) [50]. The instances of myocarditis at autopsy have been significantly higher, reaching 2-7%, than the clinical diagnosis rate [48, 51].

Electrocardiography, echocardiography, and cardiac magnetic resonance imaging are valuable tools in the diagnosis of acute myocarditis [52]. Echocardiography in patients with AM shows cardiac chamber dilatation, local or diffuse left and right ventricular dysfunction, and valvular regurgitation, possibly accompanied by varying degrees of pericardial effusion [53]. In addition to structural abnormalities, such as dilated cardiac chambers and pericardial effusion, minor symptoms, including myocardial edema, necrosis, and late gadolinium enhancement have also been detected by Magnetic resonance imaging (MRI) [48, 54]. In addition, endomyocardial biopsy has also proved to be helpful in the diagnosis of acute myocarditis; however, given the low incidence of AM, the use of endomyocardial biopsy for the diagnosis of myocarditis is not currently advocated [55].

AM often occurs secondary to a strong immune-inflammatory response after the COVID-19 infection; however, it may not be related to the direct invasion of the heart by COVID-19. The densities of CD68+ macrophages and CD3+ lymphocytes have been reported to be relatively high in AM endomyocardial biopsies, with myocardial macrophage and lymphocyte densities displaying positive correlation with the symptom duration of AM [56]. However, in cases of cardiac infection, only a small number of cardiomyocytes containing viral particles have been observed, with a median density of 1 cell/cm^2 ^[56]. SARS-CoV-2 was not detected in any of these cases. Lindner et al. compared 15 patients with undetectable SARS-CoV-2 genomes in their hearts and 16 with viral loads >1,000 copies/µg RNA in their hearts and found no differences in inflammatory cell infiltration or leukocyte counts [57]. Therefore, direct infection of cardiomyocytes with COVID-19 does not cause an obvious inflammatory response, and AM is highly likely to be secondary to a systemic inflammatory storm caused by the COVID-19 infection.

Increased epicardial and pericardial thickness can be observed on echocardiography in patients with AM and has been attributed to increased epicardial adipose tissue (EAT), a highly inflammatory reservoir with dense macrophage infiltration and increased levels of pro-inflammatory cytokines, such as interleukin 6 (IL-6) [58]. Given that EAT has direct anatomical and functional contiguity with the myocardium, Malavazos et al. considered that EAT may be implicated in the pathology of COVID-19 myocarditis; however, this requires further investigation [59].

### 1.5 Heart failure (HF) and COVID-19

HF is the end-stage state of almost all cardiac diseases and is mainly diagnosed by clinical manifestations, echocardiography, and N-terminal prohormone of brain natriuretic peptide (NT-proBNP) [60]. HF occurs in 10% of patients with COVID-19, with incidence ranging from 25% to 35% in hospitalized patients [6]. Patients with HF are highly susceptible to COVID-19. Once infected, they tend to have a more severe clinical course. Among people infected with COVID-19, diseased had a much higher proportion of HF than survivors. NT-proBNP is frequently elevated in patients with COVID-19 [61]; such patients on admission have frequent bleeding, arrhythmias, and HF decompensation [61]. Among patients hospitalized with COVID-19, those with HF (24.2%) died during hospitalization, whereas the mortality rate of patients hospitalized with acute HF was 2.6%. After adjusting for clinical variables related to COVID-19 and HF severity, the increased risk of death associated with HF history remains significant [62]. A recent cohort study spanning 18 countries demonstrated an association between the cardiac subtype and in-hospital mortality, with a considerable heterogeneity among the intensities. Cardiac patients with pre-existing HF are at the highest risk of death when hospitalized for COVID-19 [63].

Patients have been delaying care for emergencies, such as exacerbations of HF, in fear of potential COVID-19 exposure. Several countries, including the United States, United Kingdom, Germany, Italy, and Australia [64-66], have found significant reductions in HF admissions (62-72%) during the COVID-19 pandemic; however, the incidence of New York Heart Association III or IV symptoms and severe peripheral edema has been high, with an increase in the 30-day emergency readmission rate and a concurrent increase in patients with HF dying at home [66].

### 1.6 Cardiogenic shock (CS) and COVID-19

CS is a critical manifestation of myocardial injury in patients with COVID-19. Elevated cardiac biomarkers are more common clinical manifestations than CS. Of the 15,208 COVID-19 admissions recorded in the American Heart Association's COVID-19 Cardiovascular Disease Registry, 1,882 (12%) had shock, of which 105 (0.7%) had CS. Compared to patients without shock, those with CS had a higher incidence of previous myocardial infarction, coronary revascularization, and heart failure, as well as abnormal chest imaging and elevated troponin, D-dimer, C-reactive protein, and natriuretic peptides on admission. The composite of in-hospital mortality, cardiac arrest, myocardial infarction, or stroke occurred in 77% of patients with CS, but only in 13% of the patients without CS [67].

Early in the COVID-19 pandemic, endomyocardial biopsy of the first patient with CS showed low-grade myocardial inflammation and viral particles in the myocardium, suggesting viremia or infected macrophages migrating from the lung; multiple subsequent case reports have also confirmed these findings [7, 68]. However, these findings cannot verify the hypothesis that the direct infection of the virus to the myocardium results in CS. At present, it is believed that large-scale cytokine storm induced by viral infection is the major cause of CS [68]. Garau et al. reported that neither the SARS-CoV-2 genome nor significant immunoreactivity for the viral nucleocapsid protein was detected in the myocardium of patients with CS. Moreover, the pathology of COVID-19-induced multisystem inflammatory syndrome in children, resembling toxic shock, showed inflammatory cells, mainly lymphocytes and neutrophils, and no typical cardiomyocyte necrosis or SARS-CoV-2 genome in myocardial tissue and peripheral blood [69]. The putative cause of multisystem inflammatory syndrome in children is the inflammatory response disorder of SARS-CoV-2 infection [70]. Moderate doses of steroids can significantly improve the shock and multiple organ dysfunction of patients [71]. Takotsubo cardiomyopathy induced by COVID-19 may manifest as CS [72].

### 1.7 Thromboembolism and COVID-19

Venous thromboembolism (VTE), including pulmonary embolism (PE) and deep vein thrombosis (DVT), is very common in individuals infected with COVID-19 [73], with an incidence of 17% for VTE, 12.1% for DVT, and 7.1% for pulmonary embolism [74, 75]. Adults with COVID-19 have shown more than 3-fold higher risk of VTE than matched controls. Compared with patients in general wards and outpatients, the incidence of VTE is higher (27.9%) in the ICU, while it could be as high as 58% in death cases [76]. Independent risk factors for VTE include increasing age, male sex, long interval from symptom onset to admission, low fibrinogen and high D-dimer levels on admission, and D-dimer increment ≥1.5 times [77]. In addition, the significantly increased activity of factor V in patients with COVID-19 has also been associated with VTE [78].

Considering the high incidence of VTE, medical institutions aggressively administer high-dose prophylaxis or anticoagulation therapy to hospitalized patients. However, despite anticoagulation, VTE has still been reported to have occurred in 23.9% of the hospitalized patients with COVID-19 (PE, 11.6%; DVT, 11.9%) [79]. These results suggest that mechanisms other than coagulation may contribute to the thromboembolic events. For example, endothelial damage that induces tissue factor and platelet activation, low fibrinolysis, and pro-inflammatory cytokines that promote microvascular damage have been implicated in the thrombotic process [80], which may have been involved in the development of VTE due to COVID-19. In addition, the anticoagulant dose should be adjusted precisely for different patients [81]. Recently, Flumignan et al. analyzed seven studies (16,185 participants) that evaluated the benefits and harms of different doses of anticoagulants in patients hospitalized with COVID-19 and found that anti-coagulants reduced all-cause mortality compared to no treatment [82]. However, compared to the low-dose regimen, although the high-dose anticoagulants reduce PE, they offer no obvious advantage in reducing all-cause mortality, deep vein thrombosis, stroke, myocardial infarction, and atrial fibrillation incidence and may increase minor bleeding in people hospitalized for up to 30 days.

## 2. Impact of SARS-CoV-2 on specific cell types

Myocardial tissue contains many cell types, including cardiomyocytes, fibroblasts, endothelial cells, and pericytes. Almost all cells in the myocardial tissue are affected by SARS-CoV-2. However, angiotensin converting enzyme-2 (ACE2) is expressed more in cardiomyocytes and pericytes than that in endothelial cells and fibroblasts [83]. In this section, we discuss the impact of SARS-CoV-2 on various cell types.

### 2.1 SARS-CoV-2 and cardiomyocytes

**Figure 2.**
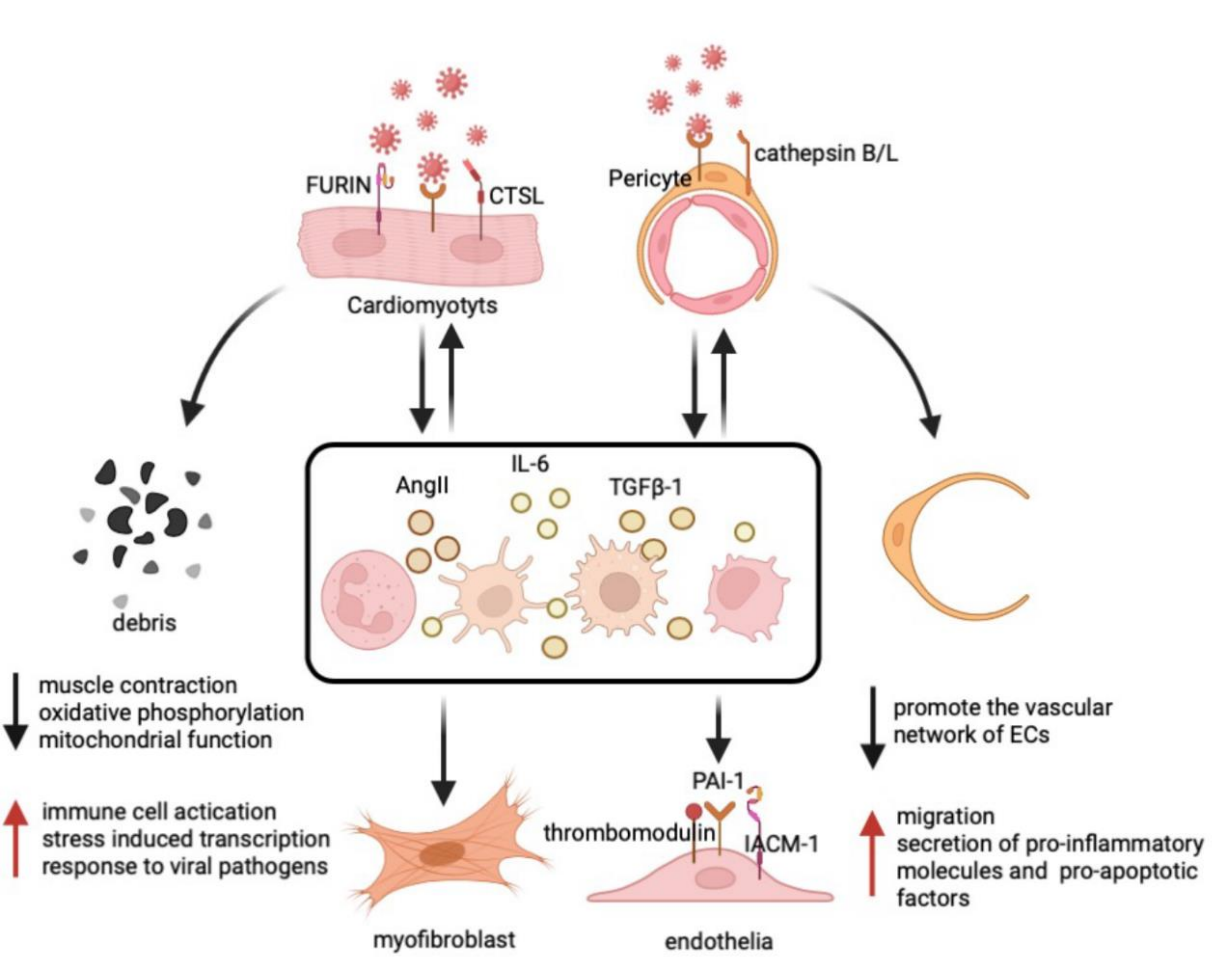
**Impact of SARS-CoV-2 on specific cell types (cardiomyocytes, fibroblasts, endothelial cells, and pericytes)**. With the assistance of CTSL and furin, SARS-CoV-2 binds to ACE2 of cardiomyocytes, leading to cardiomyocyte death, and promotes the expression of inflammatory factors (AngII, IL-6, and TGFβ). Similarly, SARS-CoV-2 infects pericytes by binding to ACE2 with the mediation of cathepsin B/L, resulting in a decrease in the formation of vascular network of ECs and an increase in the expression of inflammatory factors. Although not attacked by SARS-CoV-2 directly, secondary inflammation affects fibroblasts and endothelial cells, which do not express ACE2.

Patients with COVID-19 admitted to the ICU usually experience concomitant myocardial injury [16]. In May 2020, Hikmet et al. determined the expression of ACE2 in cardiomyocytes based on immunohistochemical analyses [84]. ACE2 was consistently expressed in the left ventricular myocardium (28.3% ± 22.2% of cardio-myocytes) [85]. In humans, ACE2 is highly expressed in the heart rather than in the lungs, whereas the S-protein promoter, protease TMPRSS2, is rarely expressed [86]. A low percentage of ACE2^+^/TMPRSS2^+^ cells in the heart tissue enhances resistance to SARS-CoV-2 up to a certain extent. However, the cellular proteases cathepsin L and furin may compensate for S protein priming to mediate SARS-CoV-2 infection in the heart [87] (Fig. 2). Given the important role of ACE2 in the process of myocardial infection, a possible strategy for COVID-19 therapy might be to inhibit the expression of ACE2 in cardiomyocytes using the recently reported miR-200c, BRD2 inhibitors, and BET inhibitors [88-90].

Myofibril fragmentation has been observed in the autopsies of patients with COVID-19, with 47 of 97 autopsies (48%) reporting SARS-CoV-2 RNA in myocardial cells [91]. Viral transcripts are present in the cytoplasm and perikaryon of the cells, which are morphologically consistent with the myocardial cells. Immunostaining of SARS-CoV-2 nucleocapsid protein showed the presence of viral protein in myocardial cells [92]. Coronavirus particles were found in hypertrophic, deformed, and necrotic cardiomyocytes surrounded by lymphocytes [93]. *In vitro* infection experiments on human induced pluripotent stem cell (hiPSC)-derived cardiac cells further elucidated the destruction process of cardiomyocytes by SARS-CoV-2; the infectious virus titer peaked on day 3 of SARS-CoV-2 inoculation into hiPSC-CMs and increased the concentrations of viral RNA in the cell supernatant [94]. Two-dimensional tissue transmission electron microscopy revealed the presence of coronavirus particles and smooth-walled extracellular vesicles containing numerous particles, sized 65-90 nm, with a typical ribonucleocapsid structure in the cytoplasm of infected hiPSC-CMs, and virus-like particles with knob-like spikes covering the cell surface [95]. In addition, SARS-CoV-2-infected cardiomyocytes stopped beating and displayed cell rounding, clumping, and syncytia formation [96]. Distortion of cardiomyocyte morphology was observed at 4-5 days after infection. On days 5-6 after inoculation, the culture medium contained a large number of dead cells and debris [97] (Fig. 2).

In hiPSC-CMs infected with SARS-CoV-2, viral transcripts accounted for approximately 88% of total mRNA [98], and host gene expression shifted from oxidative to glycolytic metabolism. Genes associated with muscle contraction, metabolism, oxidative phosphorylation, and mitochondrial function were downregulated, including components of the electron transport chain, metabolic enzymes, and cardiac contraction-related proteins, limiting energy in the myocardial cells and weakening the contractions. In addition, genes associated with immune cell activation, stress-induced transcription, and response to viral pathogens were upregulated [99], including IFNB1 and interferon (IFN)-stimulated genes, early response genes (FOS), cytokines (tumor necrosis factors (TNFs), IL1B, IL6, and CSF3), and chemokines (CCL3, CCL4, CCL7, CCL8, and CXCL8), which induce inflammation and immune damage [97] (Fig. 2). Furthermore, it was recently demonstrated that TNF-α, which is upregulated by SARS-CoV-2, promoted the expression of ACE2 and TMPRSS2, further promoting the infection of cardiomyocytes by SARS-CoV-2 [100].

Notably, the above studies are based on iPSC-derived cardiomyocytes. Although SARS-CoV-2 RNA and viral proteins were found in autopsy, it is unclear whether the virus can directly invade adult cardiomyocytes.

### 2.2 SARS-CoV-2 and pericytes

Pericytes are perivascular cells located on the near-luminal surface of blood vessels [101]; they tightly combine with endothelial cells and are mainly distributed in capillaries and post-capillary venules [102] (Fig. 2). Pericytes are the second-most common type of non-cardiomyocyte cells in the heart. The ratio of endothelial cells to pericytes is approximately 2:1-3:1 [103]. Pericytes are essential for maintaining microvascular integrity [104], coagulation homeostasis [105], and immune function. Any perturbation in the pericyte functional network, even at subdetectable levels, may permanently affect the tissue, altering vascular tone and function [106]. However, the role of pericytes in the vascular pathophysiology has long been underestimated.

Molecular analysis of the single-cell atlas of the adult heart shows that pericytes are the predominant cells expressing ACE2. In addition, they co-express cathepsin B/L protease, which functions similar to TMPRSS2, with both cleaving the SARS-CoV-2 spike protein to facilitate viral infection [107]. Therefore, pericytes are likely the main target of SARS-CoV-2 when attacking the heart. Autopsy analysis of the entire coronary tree (major coronary arteries, epicardial arterioles/venules, and epicardial capillaries) in patients with COVID-19 revealed marked endotheliitis in capillaries and high ACE2 expression, whereas the major coronary arteries showed only mild intimal inflammation [108]. This difference in intimal inflammation between capillaries and arteries is most likely due to the different distributions of pericytes. Significant reductions in pericytes and apoptosis of pericytes (cleaved caspase 3 immuno-staining) have been observed in the heart of patients with COVID-19 [109]. Moreover, SARS-CoV-2 can infect human vascular organoids, with pericytes as the main target [110]. Recent studies have confirmed that *in vitro* exposure of primary human cardiac pericytes to SARS-CoV-2 wild-type strains or alpha and delta variants resulted in infection, whereas exposure to recombinant S protein alone altered signaling and function, including: (1) increased migration, (2) impaired capacity in promoting the vascular network of endothelial cells (ECs), and (3) increased secretion of proinflammatory cytokines (IL-1 β, IL-6, TNF- α, and MCP-1) and pro-apoptotic factors (Fig. 2). In addition, immunoreactive S protein was detected in the peripheral blood of patients with COVID-19 [111].

Although the above evidence suggests that pericytes may be the target cells of SARS-CoV-2, no SARS-CoV-2 virus protein or RNA has been found in pericytes in autopsy, and there is no direct evidence of SARS CoV-2 infecting adult pericytes.

### 2.3 SARS-CoV-2 and endothelial cells

Although patients with COVID-19 exhibit marked endothelial dysfunction [112], SARS-CoV-2 is unable to directly infect ECs derived from the lung, heart, brain, umbilical vein, or kidney tissue [113]. Autopsies of patients with COVID-19 showed that SARS-CoV-2 antigens co-localized with alveolar epithelial cells and vascular smooth muscle cells, but not ECs [113]. On exposure to the virus, ECs did not undergo morphological changes, the SARS-CoV-2 nucleocapsid protein could not be detected in ECs, and viral nucleic acid was not detected in the cell supernatants. SARS-CoV-2 did not infect ECs in 3D vessels [114]. ECs are infected only when exposed to very high concentrations of SARS-CoV-2. The resistance of ECs to SARS-CoV-2 may be due to the lack of ACE2 [115]. RNA-seq analysis performed on ENCODE data of ECs from arterial, venous, and microvascular beds showed minor ACE2 expression in ECs. Primary human ECs cultured *in vitro* also lack the ACE2 receptor [113].

Although ECs express low levels of ACE2 and are resistant to SARS-CoV-2 viral infection, they are affected by COVID-19 infection [116]. MMP-1 enzymatic activity and multiple EC activation markers (soluble forms of CD146, intercellular adhesion molecule 1 (ICAM-1), and vascular cell adhesion molecule 1) were found to be significantly elevated in ICU patients [117], compared to non-ICU patients with COVID-19. The circulating endothelial cell count, a marker for assessing the severity of endothelial damage in patients with COVID-19 in the ICU, was significantly higher in ICU patients than that in non-ICU patients [118]. Thrombosis and endotheliitis are common in patients with COVID-19 [41, 108]. It is generally accepted that endothelial injury may be secondary to infection of adjacent cells and/or other pathophysiological changes, including the activation of immune cells, platelets, and complement, as well as circulating pro-inflammatory cytokines [119]. In the early stage of the pandemic, Rauch et al. used the plasma of patients with COVID-19 to culture ECs and found that the plasma significantly reduced the activity of ECs. The plasma from ICU patients had the most obvious cytotoxicity, which may be related to the increase in soluble plasma IL-2 receptors and TNF-α [119, 120]. Human ECs exposed to supernatants of SARS-CoV-2 spike protein-expressing cells developed marked cellular senescence with the expression of a senescence-associated secretory phenotype [121]. When adjacent cells are infected, ECs express increased ICAM-1, which promotes the release of pro-inflammatory factors, coagulation cascade activator plasminogen activator inhibitor-1 (PAI-1), and shedding of thrombomodulin [114, 122] (Fig. 2). In myocardial tissue, pericyte-specific and highly expressed ACE2 spreads outside endothelial cells of capillaries and venules, which may play an essential role in endothelial injury [123]. Notably, Domizio et al. constructed a lung-on-chip model and found that SARS-CoV-2 activated cGAS-STING signaling in ECs by promoting mitochondrial DNA release, leading to EC death [124].

### 2.4 SARS-CoV-2 and fibroblasts

Fibroblasts are abundant and widely distributed in the heart tissue [125]. When exposed to damage, including mechanical overload and oxidative stress, fibroblasts are activated by various cytokines and growth factors, such as angiotensin II (Ang II) and transforming growth factor-β 1 (TGFβ-1), and differentiate into an activated phenotype (myogenic fibroblasts) that promotes the synthesis of collagen and other extracellular matrix components [126, 127]. Continuous activation of fibroblasts leads to excessive synthesis of collagen and deposition of myocardial interstitial components, eventually leading to fibrosis [128]. Local cardiac fibrosis and excessive cardiac interstitial deposition have been observed in patients with COVID-19. As fibroblasts in healthy hearts barely express ACE2, which is a well-known target of COVID-19, cardiac fibrosis in patients with COVID-19 may largely be attributed to underlying diseases, such as hypertension [86].

Interestingly, recent research based on single-cell profiling analysis has reported that cardiac autopsy tissue samples from patients with COVID-19 showed a marked upregulation of cellular differentiation in fibroblasts [129]. Additionally, adventitial fibroblasts exhibit highly pronounced inflammation after short-term exposure to SARS-CoV-2 protein [130]. Although ACE2 is not expressed in fibroblasts of healthy hearts, it is expressed in more than 50% of fibroblasts in the hearts of patients with advanced HF [86]. Therefore, COVID-19 has the potential to induce ACE2 expression in fibroblasts and exert pathological effects on cells. Moreover, the levels of various chemokines and cytokines, including Ang II, IL-6, and TGFβ-1, are known to increase in the circulatory system post COVID-19 infection [131]. These factors accelerate the differentiation of fibroblasts into myofibroblasts (Fig. 2).

## 3. Cardiovascular risk after recovery

Many patients with COVID-19 display persistent dyspnea and abnormal cardiac magnetic resonance (CMR) imaging after recovery, leading to myocardial-related injury post COVID-19 recovery [132]. Myocardial injury-related symptoms (exercise-induced dyspnea, chest pain, chest tightness, and palpitations) were evident in patients with elevated troponin levels or associated cardiovascular disease [133]. Troponin levels, which represent the severity of myocardial injury, predict the occurrence of acute sequelae of COVID-19 during hospitalization [133]. Fayol et al. analyzed 48 patients with myocardial injury in the acute phase of COVID-19 and found that 60.4% of the patients still had clinical symptoms of myocardial injury at a 6-month follow-up. Low-level exercises (25 W for 3 min) induce a significant increase in the average E/e′ ratio (10.1 ± 4.3 vs. 7.3 ± 11.5, P = 0.01) and the systolic pulmonary artery pressure (33.4 ± 7.8 vs. 25.6 ± 5.3 mmHg, P = 0.02) in patients with myocardial injury during the acute COVID-19 phase. Among 148 patients with severe COVID-19 infection and elevated troponin levels, 54% (80) were found to have late gadolinium enhancement and/or ischemia during recovery stages, including myocarditis-like scars in 26% (39), infarction and/or ischemia in 22% (32), and dual pathology in 6% (9). For cases without left ventricular dysfunction, myocarditis-like lesions were limited to three or fewer myocardial segments, of which 30% had active myocarditis [31]. Echocardiographic global longitudinal strain abnormalities persisted in more than 24% of the 52 patients with COVID-19 within 6 months after discharge from the Maastricht-ICU. More than 50% of the 50 patients who recovered from COVID-19 had reduced right ventricular ejection fraction [134]. Chaturvedi et al classified 1,000 patients with COVID-19 into mild, moderate, and severe according to preexisting cardiovascular disease and the degree of COVID-19 infection and found that moderate to severe cases showed extreme cardiac damage than mild COVID-19 cases, with progressive decline in cardiac function over 3 months (LVEF -1.1 ± 0.3 vs. -3.8 ± 0.3%; mitral E/e' +3.2 ± 0.1 vs. +4.8 ± 0.1; tricuspid Vmax + 0.3 ± 0.1 vs. +1.0 ± 0.1 cm; TAPSE -0.7 ± 0.2 vs. -2.7 ± 0.2 mm (P < 0.001)) [135].

Cardiac structure (left ventricular volume, mass, and atrial area), function (ejection fraction, global longitudinal shortening, and aortic dilation), tissue characteristics (T1, T2, extracellular volume fraction mapping, and late gadolinium enhancement), and biomarkers (troponin, N-terminal pro-B-type natriuretic peptide) in patients with mild symptoms (troponin-negative, asymptomatic, or mildly symptomatic) after recovery from COVID-19 were not significantly different from those in COVID-19 seronegative healthy controls [136]. In 19 children with pediatric inflammatory multisystem syndrome after COVID-19, no persistent cardiac changes were observed on cardiac MRI during follow-up. Left ventricular size and function were normal in all patients, with normal pre-contrast T1 and T2 diastolic times, and no sign of late gadolinium enhancement. Only three children had persistent mild pericardial effusion (8-9 mm) [137]. Serious concerns have been raised on the possibility of myocarditis in athletes who have recovered from SARS-CoV-2 infection as the CMR imaging of patients hospitalized with COVID-19 suggested that they might have long-term cardiac sequelae. Several studies focusing on heart problems among athletes have shown that most were asymptomatic or had mild symptoms after contracting COVID-19. Although the incidence of abnormal late gadolinium enhancement in athletes was 2.8-38%, of which 1.4-15% met the criteria for myocarditis, that of myocardial injury is generally considered to be low (~0.6-0.7%). Athletes without comorbidities. who recovered from COVID-19. showed no signs of cardiac problems on CMR imaging [138]. In a study on young athletes infected with SARS-CoV-2, Cavigli et al. showed that only 3.2% of 571 athletes developed cardiac problems after recovery, of which more than 80% had a small amount of pericardial effusion [139]. They also highlighted the low prevalence of cardiac injury in athletes following asymptomatic or mild SARS-CoV-2 infection and did not recommend systematic echocardiographic screening for recovered athletes.

Based on a cohort of 153,760 US veterans who survived the first 30 days of COVID-19 and two control groups, a contemporary cohort consisting of 5,637,647 users of the US Veterans Health Administration (VHA) system with no evidence of SARS-CoV-2 infection and a historical cohort (predating the COVID-19 pandemic) consisting of 5,859,411 non-COVID-19-infected VHA users in 2017, a study highlighted the effects and prevalence of COVID-19 [140]. After adjustment for factors, such as age, race, sex, and other cardiovascular risk factors and the initial 30 days of infection, patients with COVID-19 exhibited an increased risk of cardiovascular diseases, including cerebrovascular disease, arrhythmias, inflammatory heart disease, ischemic heart disease, heart failure, thromboembolic disease, and other cardiac diseases. In addition, the risk of cardiovascular disease after COVID-19 was also evident in patients without any history of cardiovascular disease [140]. Similarly, Singh et al. reported that recovered patients with COVID-19 had cardiac dysfunction irrespective of COVID-19 disease severity, presence of myocardial injury, or ongoing symptoms [141]. The above two studies highlight the high cardiovascular risk among COVID-19 survivors, suggesting that cardiovascular health care strategies must be developed for survivors after acute COVID-19 exacerbation.

The mechanism of the relationship between COVID-19 and cardiovascular disease after acute infection remains to be clarified. One possible explanation is that there is still potential chronic low-grade inflammation in the heart after COVID-19 infection [142]. In asymptomatic and moderately infected patients, significant residual inflammatory reaction can be observed 40-60 days after virus infection [143]. Long COVID (LC) is defined as ongoing symptoms of COVID-19 that persist beyond 4 weeks from initial infection. Patients with LC are more likely to have chest pain. The long-term elevation of cardiac troponin in patients with LC is significantly related to the increase in mortality [144]. Compared to healthy people, patients with LC have highly activated congenital immune cells, lack naive T and B cells, and show a sustained high level of type I IFN (IFN- β) and type III IFN (IFN- λ1) at 8 months after infection [145]. Therefore, it is currently believed that regulating immunity and anti-inflammation can be the treatment direction for LC. Vitamin D [146], vitamin C [147], and rosemary [148], which have anti-inflammatory and immunomodulatory effects, have been proved to alleviate LC. Chronic low-grade inflammation may be caused by SARS-CoV-2, which destroys the immunity of individuals, resulting in failure to completely remove the virus, persistence of residual virus, and increased release of pro-inflammatory cytokines. In addition, the reactivation of EB Virus after COVID-19 infection may also be one of the causes of chronic low-grade inflammation [149].

In addition to chronic low-grade inflammation, vascular endothelial injury [149], microvascular thrombosis [150], and imbalance of the renin angiotensin aldosterone system and kallikrein releasing enzyme system [151] are also important mechanisms leading to myocardial tissue damage. Further investigations on the biological mechanisms are required to support preventive and therapeutic strategies for cardiovascular disease in patients with COVID-19.

## 4. New problems posed by vaccines

Vaccination is the cornerstone of preventing SARS-CoV-2 infection. As of August 1, 2022, 12,308,330,588 vaccination doses have been administered. Adenoviruses are the backbone of traditional vaccines and can easily produce antigenic epitopes with stable and appropriate conformation [152]. Given their rapid production and the generation of various potentially immunogenic proteins by antigen-presenting cells, messenger RNA vaccines are currently the primary vaccines administered worldwide [153]. Vaccination against COVID-19 alleviates fears of SARS-CoV-2 infection but is persistently associated with adverse effects (such as myocarditis/pericarditis, deep vein thrombosis, disseminated intravascular coagulation, and encephalomyelitis) [154-157], causing hesitancy among the public, thus influencing the medical decisions to a certain extent; for example, in Russia, only 55% of the general population is willing to be vaccinated against COVID-19 [158, 159].

Myocarditis and pericarditis are the most common COVID-19 vaccine-related cardiovascular side effects. Of the 716,576 reports associated with the side effects of mRNA COVID-19 vaccine, 2,277 involved inflammatory cardiac reactions, including 1,241 (55%) of myocarditis and 851 (37%) of pericarditis [160]. The onset of COVID-19 vaccine-associated myocarditis/pericarditis usually occurs within a week of vaccination and manifests as an acute onset of dyspnea, palpitations, or acute chest pain. Diagnosis is based on myocardial injury markers, electrocardiogram, echocardiography, or cardiac MRI in combination with the patient’s clinical presentation [161-163]. A survey of more than 40 million vaccinated individuals in England found that the overall incidence of COVID-19 vaccine-related myocarditis was 10-60 cases per 100,000 people. The vaccine-related increased risk of myocarditis most often occurs after the second dose of vaccine in men aged 12-29 years [164, 165]. Although the proportion of myocarditis associated with COVID-19 vaccine is relatively high, most cases are mildly symptomatic, and >90% of patients fully recover their cardiac function after prompt treatment [166].

COVID-19 vaccine-associated reversal of Takotsubo cardiomyopathy, catecholamine-like stress (toxic) cardiomyopathy, cytomegalovirus reactivating peri-carditis, and fatal fulminant necrotizing eosinophilic myocarditis have also been reported [167-170]. The diversity of myocardial injury caused by the COVID-19 vaccine suggests the importance of the genetic background of immunity and prompts researchers to continuously adjust the vaccine strategy to reduce adverse effects. Certain vaccine components and SARS-CoV-2 viral proteins molecularly mimic molecules in cardiac tissue; this is currently considered the main cause of vaccine-related myocardial injury. Polyethylene glycol in mRNA vaccines is a recognized sensitizing excipient that may cause allergic reactions and induce secondary myocarditis; however, the sensitization mechanism of polyethylene glycol is still unclear [171]. Antibodies against SARS-CoV-2 spike glycoprotein may cross-react with structurally similar human protein sequences, including the cardiac α-myosin heavy chain, causing autoimmune myocarditis [172]. In addition, the vaccine has been reported to potentially increase the load on the heart, causing relative ischemia and leading to myocardial damage.

The incidence of COVID-19-related myocardial injury (1,000-14,000 per 100,000 people) is approximately 100 times that of vaccine-related myocardial injury [173]. COVID-19-related myocardial injury mainly occurs in advanced ages and in the presence of preexisting conditions that can cause myocardial damage (diabetes, high blood pressure, and renal insufficiency). The COVID-19 vaccine has shown promising results with a 1,000-fold reduction in the risk of myocarditis/pericarditis in the general Israeli population within the first 42 days after the first dose of Pfizer-BioNTech mRNA vaccine [174]. Therefore, despite the adverse effects of current vaccines, after weighing COVID-19 complications and vaccine-related risks, medical experts unanimously recommend vaccination in adolescents and adults to prevent heart problems caused by COVID-19 [172].

## 5. Conclusion and remarks

The COVID-19 pandemic has had a devastating impact on a global scale. The direct and indirect effects of SARS-CoV-2 on cardiomyocytes, pericytes, fibroblasts, and endothelial cells explain the various cardiovascular manifestations caused by COVID-19 at the cellular level, ranging from cardiac troponin increases to severe cardiogenic shock. In the post-pandemic era, vaccination-induced myocardial injury and long-COVID-19 are key issues in the cardiovascular field, and the related mechanisms require further investigation. In the past 3 years, researchers have discussed the above problems and phenomena in detail; however, the following problems still persist and require further studies.

Pericytes may be the main target of SARS-CoV-2 attacking the heart; however, the role of pericytes in SARS-CoV-2-induced vasculopathy has been neglected. To the best of our knowledge, the presence of intimal inflammation in patients with COVID-19 may be attributed to pericyte infection by SARS-CoV-2; however, the exact role of pericyte damage in the progression of COVID-19 remains unclear. The specific effects of pericyte damage, including their participation in cytokine storm, vascular structural changes, and microthrombosis need to be clarified in order to identify the role of pericytes in COVID-19. ECs and fibroblasts scarcely express ACE2 but are still affected by SARS-CoV-2, as evidenced by microcirculatory disturbances and myocardial fibrosis. Elevated Ang II and IL-6 in the circulation may partially explain this phenomenon, but more basic research is required to reveal the underlying mechanism.

The cardiovascular symptoms caused by SARS-CoV-2 include acute myocardial injury, arrhythmia, acute myocarditis, and heart failure [175]. In addition to the damage caused by the direct invasion of myocardial tissue by SARS-CoV-2, immune-inflammatory responses play a prominent role. Epicardial adipose tissue, which shares microcirculation with myocardial tissue, is rich in pro-inflammatory cytokines and plays an important role in the immune inflammatory storm induced by SARS-CoV-2; however, the role of epicardial adipose tissue still needs to be carefully verified. VTE induced by COVID-19 can hardly be prevented, and the efficacy of anticoagulant therapy is limited, suggesting that future clinical studies are needed to clarify the use of anticoagulants. Causes and mechanisms of thromboembolism other than coagulation should be further explored based on basic research. The implementation of the COVID-19 lockdown policy and the fear of contracting COVID-19 have led to a significant drop in hospital visits for cardiovascular disease (such as heart failure and acute myocardial infarction) and a parallel increase in mortality and related complications. During the COVID-19 pandemic, additional deaths due to nonbiological factors have prompted reforms in medical advocacy, services, and management.

LC has drawn attention to myocardial injury after recovery, which has become an important topic in the post-pandemic era. Newly-emerging symptoms (e.g., post-COVID-19 tachycardia syndrome) suggest that the assessment and long-term monitoring of long-COVID and pathogenesis are important areas for further research. In addition, with the expansion of the vaccinated population, vaccine-related myocardial injury has attracted increasing attention; however, its pathogenic mechanism is still not fully understood. Reducing the sensitization of the vaccine and the cross-reaction between vaccine components and structurally similar human protein sequences also requires further research. Vaccination has dramatically reduced the proportion of critically ill patients; however, infection prevention and control measures, such as social distancing and personal hygiene, remain the cornerstones of managing this global pandemic. Vaccine development and ongoing research on SARS-CoV-2 reflects the tireless efforts of the scientific community, healthcare workers, and government agencies. The lessons learned from our response to COVID-19 will hopefully prepare us for future pandemics.
